# Multielemental Analysis of Various Kinds of Whisky

**DOI:** 10.3390/molecules24071193

**Published:** 2019-03-27

**Authors:** Aleksandra Pawlaczyk, Magdalena Gajek, Krzysztof Jozwik, Malgorzata Iwona Szynkowska

**Affiliations:** 1 Institute of General and Ecological Chemistry, Lodz University of Technology, Zeromskiego 116, 90-924 Lodz, Poland; magdalena.gajek@edu.p.lodz.pl (M.G.); malgorzata.szynkowska@p.lodz.pl (M.I.S.); 2 Institute of Turbomachinery, Lodz University of Technology, Wolczanska 219/223, 90-924 Lodz, Poland; krzysztof.jozwik@p.lodz.pl

**Keywords:** whisky, multielemental analysis, ICP-MS, mercury analyzer, chemometric analysis, PCA

## Abstract

Whisky (whiskey) consists of many trace elements coming from the raw materials used in its fermentation, distillation and maturation processes. These ingredients assure the exceptional organoleptic characteristics of the beverage. Their analysis is important to better control the stages of fermentation, distillation, taste repeatability and for product quality assurance as well as from the brand protection point of view. This article presents the usefulness of modern analytical techniques based on elemental analysis. ICP mass spectrometry and CV atomic absorption spectroscopy were applied to distinguish whisky produced in Scotland from whisky coming from Ireland and the United States. The collected semi-quantitative data were used for multivariate analysis performed using the Statistica 10.0 software. The results showed that Irish whiskey is characterized by quite a high amount of Ba and Ti compared with other samples, which made it possible to distinguish this sample from the others. No strict correlation was found between the type of whisky and the amount of trace elements, however, the projection of objects on the first two components revealed that single malt samples created one cluster.

## 1. Introduction

At present, whisky (the alternate spelling whiskey is commonly used in Ireland and the USA–for consistency the former spelling is used in this paper) is one of the most popular alcoholic beverages in the world. According to the report of the IWSR company and the Just-Drink portal, the consumption of Scotch whisky in the world increased by 193.5 million liters between 2001–2011, which could be compared to the appearance of a completely new country inhabited by people only drinking whisky.

According to the current definition, whisky is a type of distilled alcoholic beverage made from fermented grain mash. A wide variety of grains is used in the production of different types of whisky, for example, barley, corn, wheat and rye. Typically, wooden casks, made of charred white oak are used in the process of whisky aging [1]. Worldwide, whisky is subjected to strict regulations with many types and classes in terms of fermentation of grains, distillation, and aging in wooden barrels.

According to the current state of knowledge, whisky originates from Ireland. This is where in the seventh century the monks in the monastery distilleries produced distilled alcohol based on herbs and essential oils known in Latin as “aqua vitae”. Initially the mixture was used for medical purposes only. In the course of the medical distillation passed from a monastic setting to the secular by doctors and pharmacists [2]. After the British invasion of Ireland in the 12th century, the recipe for “aqua vitae” reached the British Isles with the changed name “uisge beatha” [3]. The first official recording of distilling when John Cor from Fife was granted the king’s commission to make “water of life” dates to 1494. Due to the high availability of grain, farmers started to deal with distillation. In 1644, the British Parliament taxed the production of this spirit. This, of course, resulted in an increase in an illegal production. In 1779, there were about eight legal and 400 illegal distilleries. At that time the commonly used term “uisge beatha” was changed into “uisge”, “usky”, and finally to “whisky”. About 30 years later, Parliament relieved restrictions on authorised distilleries, thereby ushering in the modern era the production of Scotch whisky [4]. Two events contributed to the increasing popularity of whisky. Firstly, the new process of production presented by Andreas Coffey in 1831 made the alcohol smoother and less intense. Secondly, the pest destroyed cultivation of grapes in France in 1880, so stocks of wines in cellars of in France and around the world declined dramatically. Since then, the production of whisky has been growing. Today, this alcohol is drunk in over 200 countries around the world. Currently there exist three types of whisky: malt whisky, grain whisky and blended whisky. It is also possible to distinguish single barrelled whisky but it is not a new type but a product of special taste of alcohol coming from one barrel not always repeatable taken into consideration another barrel. Blended is the most popular type available on the market, produced via mixing malt and grain whisky. Single malt is divided into single cask, cask strength and pure malt, which differ from each other in the distillation alcohol percentage, and the origin of the malt. Whisky grain is produced from a mixture of different cereal species (barley, wheat and maize). In its pure form, single grain whisky is rarely seen. It is mainly used as a component of coupage in the production of blended whisky. The production of whisky should take place under strict conditions. The aging process lasts a minimum of 3 years in an oak barrel, or 2 years in the case of American bourbon [5].

### 1.1. Whisky in the World

In majority of countries, the law regulates the definition of whisky. This ensures protection of the producer, consumer, and tax revenues for the state. If a country is not a whisky producer, the definition of the home country applies. The regulations for the country of origin are presented in the later part of the article.

#### 1.1.1. Scotch Whisky

Scotch whisky, both malt and grain, is produced only in Scotland based on local cereals, water and yeast. Malt whisky is produced from malted barley. The next step is concentration of the alcohol during the distillation process in a copper alembic. Grain whisky is produced from non-malted cereals, such as wheat, maize, or barley and small part of malted barley. In the next stage, in the distillation process the alcohol is also concentrated. The Coffey Still remains the most popular procedure. In both cases, alcohol content is reduced by adding water from 65 to 70%. Scotch whisky must be matured in oak casks for at least three years. After that, whisky may be used as a single product or blended with others, and again the alcohol content is lowered by water to a minimum 40%. The last step is bottling [6]. Current law defines five categories of Scotch whisky:

(1) Single Malt Scotch Whisky,
(2) Single Grain Scotch Whisky,
(3) Blended Malt Scotch Whisky,
(4) Blended Grain Scotch Whisky,
(5) Blended Scotch Whisky.

According to the Scotch Whisky Association, currently Scotland is divided into five regions: “*Speyside*”, “*Lowland*”, “*Highland*”, “*Campbeltown*” and “*Islay*” [7].

#### 1.1.2. Irish Whiskey

Legal definitions for Irish whisky can be found in the “*Irish Whiskey Act 1980*”. Additionally, the product is protected by European Geographical Indication in accordance with Regulation (EC) No 110/2008. The most important requirements for Irish whisky are as follows:

- The alcohol must be produced in Ireland from grain malt with or without mixtures of unmalted and malted cereals,
- Irish whisky must be triple distilled (three pot stills for malt whiskey; continuously distilled with three-column systems for grain whiskey),
- The only addition to the distillate is water and dye E150a—caramel,
- The minimum alcohol volume content must be 40%,
- The maturation process, for a minimum of 3 years, takes place in Ireland,
- The term ‘single’ may refer only to a distillate from one distillery [8].

The number of distillations is the basic difference between Scotch and Irish whisky. It is double for Scotch and triple for Irish whisky. The possibility of addition of exogenous amylolytic enzymes in the mashing process in the case of Irish whisky is another difference [4].

#### 1.1.3. American Bourbon 

According to the Federal Standards of Identity for Distilled Spirits, U.S. whisky:

- Has to be distilled in the United States,
- Has to consist of a mixture of grains (at least 51% corn),
- Has to be matured in new, charred casks,
- Has to contain at most 80% pure alcohol after distillation,
- Not be poured into barrels if it contains more than 62.5% pure alcohol,
- Has to be aged less than four years (e.g., 2 years for the European market) and include information about the period of maturation [3,9].

#### 1.1.4. Other Countries

Other countries also apply strict regulations on the production of whisky. Countries like Japan, Australia and New Zealand require whisky to be made from cereal grains. In Canada whisky can be obtained from a mash of cereal grain or cereal grain treated with enzymes. Moreover, the product must be aged for at least three years. The addition of caramel and flavours is allowed.

### 1.2. Product Authentication

Counterfeiting of products, such as food, cosmetics and medicines, is nowadays a common problem on a global scale and can be dangerous to the life and health of consumers. Dynamic economic development and an increase in the standard of living resulted in a growing interest in brand products, which opens up possibilities of generating considerable revenues from counterfeiting products. It is estimated that around 9% of all global trade comes from the trade of counterfeit goods. Some of the main product groups with the largest upward trend in the number of counterfeit products are drugs, cosmetics, honeys, wines and other alcoholic beverages. A possible solution to this growing problem is product authentication, which is a process of verifying the identity of the declared product. In the beginning the trace elements analysis of different alcohols was carried out in order to assess e.g., the possible toxicity of the studied products. The information regarding the concentration of specific elements, like heavy metals, was crucial in terms of the unfavourable impact they may have on consumers and the organoleptic properties of alcohols like wines. In 2004, Kim investigated sixty samples of red and white wine purchased from local shops in Korea and originating from different counties, including France and Australia. Even though some differences in the content of Pb and Cd were found between the red and white wines, no significant variability of the obtained data was noticed in relation to the country of origin. According to the author, the non-conclusive results may arise from both the natural (e.g., such as soil composition and grape variety) and exogenous factors (like the fermentation process, wine-making system or from different kinds of contamination, including applied pesticides or fertilisers) [10]. Since then many papers have been published in which the possibility of differentiation of various alcohols (mainly wines) based on the region of their production was presented. Some reports clearly suggest that the elemental composition is highly influenced by the geographic origin of the grapes. Xue at al. investigated the content of the following elements: As, Ba, Co, Ni, Cu, Be, Sr, Cr, Mn, Li, P, Se, Pb, Bi, Cd, B, Zn, Mg, Fe, K, Na, Ca and Al in 19 wines produced in Jiangxi, China, in terms of their potential differentiation based on producing regions, alcoholic degrees and qualities from the same manufacturer. The results proved that there is some correlation between the quality of wine and the trace element composition, since the wines produced from different regions and with different alcoholic degrees and qualities from the same manufacturer clearly indicated differences among themselves [11]. The practical fingerprinting of wines was also performed by Płotka-Wasylka et al., where the wine samples were readily separated by geographic objectives, and for each region some discriminating metal variables were defined [12,13]. In another work, the wine samples were classified according to the geographical origin of the grapes. In this study an elemental composition of these samples was used to check a classification model that discriminates Chianti from other wines [14]. In the review paper by Wiśniewska et al., a comprehensive summary concerning analytical studies of the whisky and other alcoholic beverages in the context of the assessment of their quality, origin, authentication and the identification, was presented. The possibility of the application of the various techniques such as atomic absorption spectroscopy, mass spectrometry or gas and liquid chromatography used to evaluate the composition of studied samples have been widely described as well. This article provides extremely valuable knowledge in the field of the authentication research of whisky and other alcoholic beverages, however, to the best of our knowledge, there are not so many detailed data regarding the elemental analysis of whisky employed for the potential classification of their origin and type [15]. In one of the rare examples of whisky studies Meier-Augenstein et al., used ^2^H and ^18^O isotopic signatures by High Temperature Conversion–Isotope Ratio Mass Spectrometry (TC/EA–IRMS) to successfully discriminate samples of authentic Scottish whisky from the samples of counterfeit whisky based on the strong correlation between the composition of the same isotopes of δ^2^H and δ^18^O in local source water used for and during whisky production and in original whisky products from Scotland [16]. In an other study the, isotope ratio ^13^C/^12^C of various volatile compounds such as e.g., N-propanol, isobutanol or ethyl acetate, amyl alcohol and acetaldehyde were applied to discriminate different types of Blended whisky. It was shown that a comparison of the isotope ratio can be successfully used to differentiate among whisky brands by gas chromatography-combustion—isotope ratio mass spectrometry [17]. Adam et al., determined the concentration of eight elements (Ca, Cu, Fe, Pb, Mg, Ni, Na and Zn) in 35 samples of Scotch whisky in order to use the gathered information (a metal fingerprint) to locate the origin of the whisky. When all elements were considered for cluster analysis, no distinctive classification was obtained. Thus, it was concluded that metals concentration fingerprint cannot be unquestionably employed as primary criteria to identify various Scotch whisky-producing regions. The same authors then focused their interest only on the copper analysis since they indicated that the whisky product has a rather unique copper concentration. It was proven that the difference between Cu level in Single Malt and Blended Scotch whisky is highly significant and can be used as an indicator to discriminate whisky by its type [18]. The content of 11 elements (P, S, Cl, K, Ca, Mn, Fe, Cu, Zn, Br and Rb) was determined with the use of the total reflection x-ray fluorescence (TXRF) technique in 25 original samples which were produced in different regions of Scotland, and in five counterfeit whiskies and in one unmatured and one matured grain whisky by Shand at al. The authors were unable to distinctively separate the samples by principal component analysis based on their region or type, however, they successfully distinguished the counterfeit samples from the other ones [19]. In an another paper, the concentration of Pb, Cd, As and Hg was evaluated in different types of alcoholic beverages, including whisky, brandy or wine, and the obtained results were compared with maximum levels given by the local food code nevertheless no discrimination or classification was undertaken [20]. Due the fact that only a few limited papers on metal analysis in whisky are available, we sought to use metal mass spectra fingerprint and principal component analysis to test the possibility of distinguishing whisky samples based on their origin and type. Thus, in this study an attempt has been made to verify if the variability of elemental composition can be correlated with the country of production. The presented results can broaden the knowledge in the area of whisky fingerprinting.

## 2. Results and Discussion

The aim of this work was to assess the potential correlation between the elemental composition of the whisky samples and the country of their origin based on the semi-quantitative data obtained by ICP-MS (21 isotopes: ^44^Ca, ^45^Sc, ^47^Ti, ^48^Ti, ^51^V, ^52^Cr, ^54^Fe, ^55^Mn, ^60^Ni, ^63^Cu, ^66^Zn, ^88^Sr, ^90^Zr, ^95^Mo, ^101^Ru, ^107^Ag, ^111^Cd, ^118^Sn, ^138^Ba, ^208^Pb, ^209^Bi and quantitative information gathered by CV AAS technique (total Hg content [µg/L]). Additionally, the chemometric methods were applied in terms of identification of similarity among objects. Finally, two main parameters were taken into account, namely the region of their origin as well as the type of product. The evaluation of the dependence of the age of the product turned out to be non-conclusive and was rejected from further analysis.

### 2.1. Mercury Analysis 

Based on the obtained results, it can be stated that whisky from Scotland has the lowest amount of Hg when compared with other samples. The Speyside turned out to be the region with the lowest traces of Hg (the average amount reached 1.82 μg/L). The highest content of Hg was detected in whisky originating from the USA, which was two and a half times higher (4.56 μg/L) than the average value measured for Scottish Speyside whisky. Highland Scottish whisky and the Irish brand whisky showed almost identical levels of mercury content of 2.13 μg/L and 2.16 μg/L, respectively. Moreover, it can be clearly seen that single malt whisky has a smaller amount of mercury, and the results for the studied population were quite comparable, while for blended whisky the median value was higher and the results especially for the highest 25% of observations were the most diversified. However, no statistically significant differences were stated for these two factors (the origin of samples and type of samples). The results were compared with the established permissible levels of mercury. According to the Polish Regulation of the Minister of Health regarding the maximum levels of chemical and biological contaminants that may be found in food, food ingredients and authorized additional substances, substances helping in survival or being on the food surface from the 13 January 2003, the permissible content of mercury in spirits containing more than 20% of alcohol is set to be 20 μg/L. In any case mercury, the content in studied whisky samples does not exceed the permissible level of 20 μg/L.

### 2.2. Mass Spectra Profiles

Mass spectra were collected for each of the whisky samples. The acquisition time was set up to be 3 s. Three replicates were saved and the average value of the signal intensity for each *c* was then used for multivariate data analysis.

**Figure 1:**
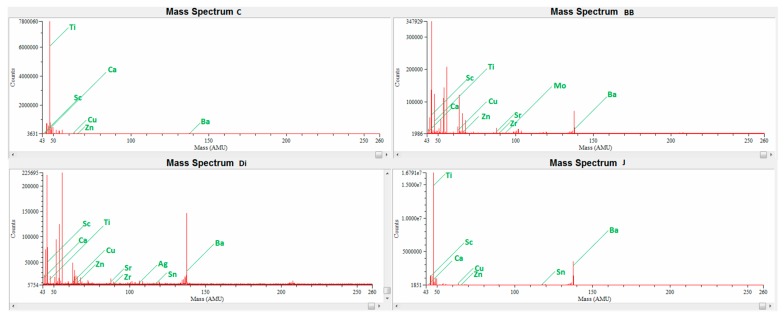
Exemplary ICP mass spectra of four samples of whisky showing characteristic fingerprint of each sample, obtained for the whole mass range from 43 to 260 *m/z*.

Examples of four mass spectra recorded for different studied samples are an evidence of a unique whisky profile. It can be concluded that the intensity of signal at selected *m/z* and proportions among analyzed isotopes can create characteristic fingerprint of each sample. All samples are presented in Figure 1. The recorded mass spectra refer to blended whisky, but sample J originates from Ireland, while samples C, BB and Di come from various regions of Scotland. Sample J is characterized by the highest amount of Ti, Zn, Sr, Zr, Sn and Ba. Sample C has the highest, amount of Ca and Sc and a relatively high amount of Ti and Mn. Considerable for sample Di is typical but not the highest amount of elements like: Cr, Mn, Ni, Cu, Ag, Ba, Pb. Sample BB contains a significant amount of Zn and Sr.

### 2.3. Data Analysis Using a Non-Parametric test

Non-parametric tests can typically test assumptions about the data and may be more relevant in many particular situations. For example, it is possible to check the hypothesis tested by the non-parametric test that may be important for the conducted research such as a possible influence of a sample is origin or type. However, the prior assumptions have to be made upfront. In addition, their interpretation is often more direct than multivariate analysis. Non-parametric statistical tests can be used to analyze the data which are inherently in ranks as well as the data whose numerical scores have the strength of ranks, without being able to say how much more or less. Thus, non-parametric methods are available to treat the data which are classificatory or categorical e.g., by the median test which is used to compare the performance of two independent groups.

➢ *For All Objects*

**Figure 2:**
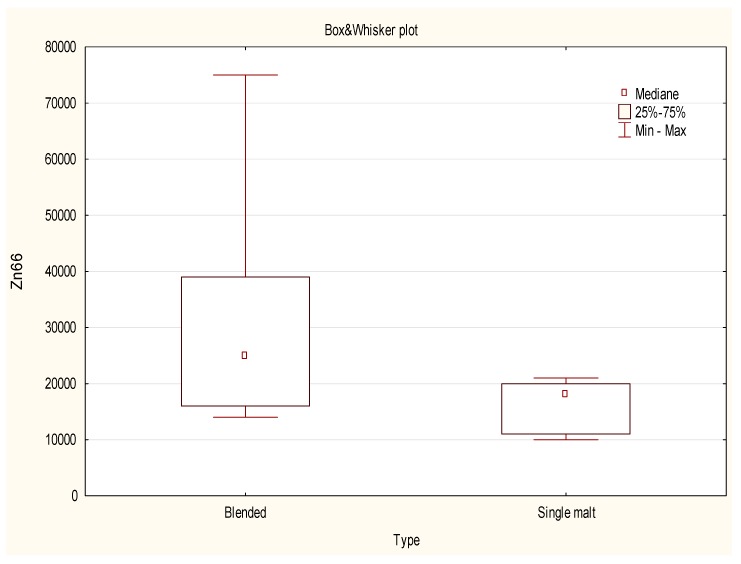
Box-Whisker Plot of Zn semi-quantitative data obtained for 20 objects (whisky samples from the USA (Blended), IRL (Blended) and SCT (Blended and Single Malt)).

**Figure 3:**
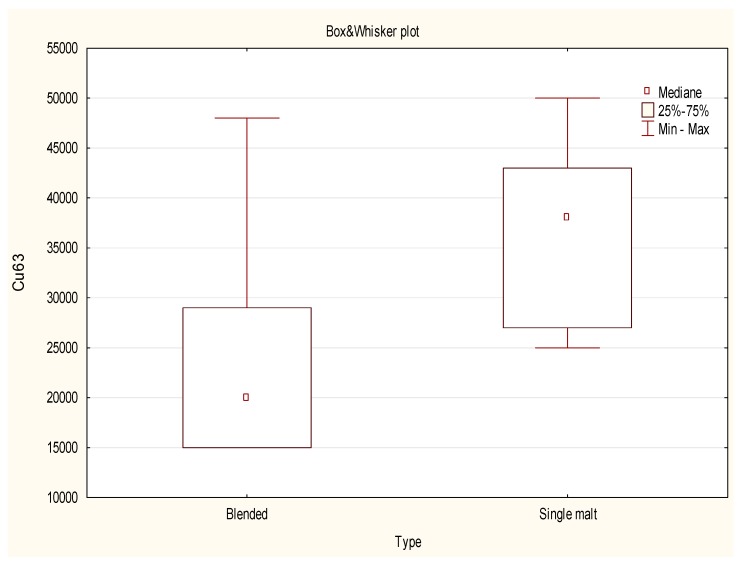
Box-Whisker Plot of Cu semi-quantitative data obtained for 20 objects (whisky samples from the USA (Blended), IRL (Blended) and SCT (Blended and Single Malt)).

A non-parametric test was finally applied to check the potential differences in the content of selected isotopes in relation to the region of origin and the type of whisky. Samples were firstly grouped into two clusters: Blended and single malt whisky. The non-parametric test showed the presence of statistical differences between the intensity of signal only for ^52^Cr, ^54^Fe, ^63^Cu, ^66^Zn and ^138^Ba. In this group, a blended type of whisky contained much more Fe, Zn (Figure 2) and Ba with a considerably lower amount of Cr and Cu (Figure 3) when compared with Single malt whisky. The origin of the traces of Cu can be the alembic, which as a rule is made of copper. This metal enters a chemical reaction with a distillate and somehow “extracts” sulfuric aromas from it. Consequently, the size of the alembic is crucial since the longer the distillate touches the copper, the softer it will be. All three parts that constitute the alembic boiler are welded together using a brass wire or other solid and non-reactive fillers. The gas welding process causes the copper to become flexible and plastic. After that, the soldered copper elements are stuck on the anvil to restore its strength and resistance. The remaining parts of the alembic are soldered either with tin or silver, whose presence was also detected by ICP-MS technique. Malt whisky is produced in the traditional copper stills in batch-type rectors, while grain whisky which, in general, contributes the most into the Blended whisky, is run continuously using more industrial style patent stills. Thus, Malt whisky being distilled in small traditional pot stills is expected to contain more copper than Blended or Grain whisky. Adam et al., also stated the whisky has a uniform copper concentration and that the mean copper concentration was significantly higher for all Malt whisky samples than for Grain and Blended Scotch whisky samples [18].

For the other isotopes for which no statistical differences were observed between both groups, the median value of the intensity of signal for ^45^Sc, ^88^Sr, ^90^Zr, ^118^Sn, ^208^Pb, ^209^Bi and Hg was higher for blended whisky. For isotopes like ^44^Ca, ^55^Mn, ^60^Ni, ^107^Ag, the median value was higher for single malt whisky. For the rest of isotopes (^47^Ti, ^48^Ti, ^51^V, ^101^Ru, ^111^Cd), the median values were almost equal in both groups. For most of the studied isotopes, the biggest diversity of 50% of the typical values was recorded for blended whisky. The diversity of the typical values was comparable for ^111^Cd within both groups, whereas for ^101^Ru, variability was more significant in the group of Single malt whisky. It is postulated that elements like Fe or Ni can be constituents of special stainless steel tubings, which can be used in specific distilleries. Moreover, it cannot be excluded that many distilleries may treat their water using ion exchange cartridges, which may affect the metal distribution of the water [18]. 

In the second step of the data analysis, the non-parametric test was employed to check a possible difference among three groups: USA, IRL, SCT in reference to their origin. Any statistically significant differences were confirmed by this test. It should be, however, underlined that two out of three groups were represented by single whisky objects. The data set was divided into two groups (Scotland and Others) and based on the results of the same test. It was shown that the statistical differences can be related to the content of three elements: Ba, Zn, Sn. For all samples belonging to the “Scotland group”, much lower median values were stated. For Ba, the obtained results in the scottish whisky were much less diversified than for other types of whisky as opposed to the results gathered for Zn. The diversity of the result for Sn was similar in both groups. Finally, all the results were divided into four groups: SCT S, SCT H, SCT L, Others. The existence of the statistically significant differences was proved for the Sn content exclusively. The highest median value was reported for “Other” group (USA and IRL), while the biggest variability of the results was revealed for the samples coming from the Lowlands region of Scotland.

➢ *After the Reduction of Objects*

Due the fact that among the investigated samples there were only two representatives of other countries than Scotland, a non-parametric test was used again, but this time only in order to study the statistical differences within various regions of Scotland (Highlands—6 objects, Lowlands—2 objects and Speyside—10 objects) for the reduced set of objects. It was shown that in relation to the origin of the sample, the statistical differences were observed only for elements such as Pb and Bi. Analysis of multiple comparisons proved that the differences between the intensity of signal for the samples originating from the Highlands and Speyside regions were responsible for the stated statistical differences. In both cases a slightly higher intensity of signal for Pb and Bi was noticed for the samples taken from the Highlands region. Moreover, both for Pb and Bi, the biggest variation of 50% of the most typical values was observed for Lowlands region. In the case of the studied remaining elements, the most often the highest median value was reported for the Highlands region (^44^Ca, ^51^V, ^54^Fe, ^55^Mn, ^60^Ni, ^66^Zn, ^88^Sr, ^90^Zr, ^95^Mo, ^118^Sn, Hg). For isotopes such as ^45^Sc, ^52^Cr, ^63^Cu, ^101^Ru, ^107^Ag, ^138^Ba, the highest value of median was stated for the Lowlands region. For this region of Scotland, the greatest diversity of the results was found the most frequently, which can be a consequence of the low number of observations belonging only to the blended type of alcohol. Even though the Speyside region was represented by the biggest amount of cases (10 whisky samples), for any of the studied isotopes the highest median value was achieved. It should be also highlighted that four out of five analyzed types of Single Malt whisky originated from this region, which can strongly contribute to the lesser diversity of the results within 50% of the typical values. For isotopes like ^47^Ti, ^48^Ti, ^111^Cd, no differences among the median value were observed for various areas in Scotland. 

For the same reduced set of objects, the hypothesis concerning the differences among type of whisky was tested. Only for two isotopes: ^54^Fe and ^63^Cu, the statistical differences between the blended whisky type (13 objects) and single malt whisky (five objects) were confirmed. In the case of ^54^Fe, the blended type contained more ^54^Fe while the opposite situation took place for ^63^Cu. It is assumed that the amount of Cu and other metals is mostly determined by the use of copper stills rather than the origin of the water or barley. We can conclude, similar to the findings by Adam at al.’s that since the differences in semi-quantitative results for Cu and Fe are statistically significant for these metals, as indicated in this study for Scottish whisky, they can all be potentially used to identify the Malt whisky from the Blended type. However, more studies are needed regarding the bigger data set, including more samples representing different regions and more samples taken within the same distillery.

Regarding other studied isotopes, a higher median value was mostly indicated for single malt whisky, like for ^45^Sc, ^51^V, ^52^Cr, ^55^Mn, ^60^Ni, ^107^Ag, ^138^Ba, ^208^Pb, ^209^Bi and Hg. For isotopes like ^44^Ca, ^47^Ti, ^48^Ti, ^88^Sr, ^90^Zr, ^95^Mo, ^101^Ru, ^111^Cd, ^118^Sn, the median values for two whisky types were comparable. It should be also noted that almost in all cases the results gathered for blended whisky showed significantly more diversity of 50% of the most typical values with the exception of ^63^Cu, ^101^Ru. For isotopes like ^47^Ti, ^48^Ti, ^54^Fe and ^111^Cd, the diversity of the most typical values was similar in both groups. It can be concluded that the highest diversity of 50% of most typical observations for blended whisky can be attributed to the fact that in this study blended whisky was represented by all three regions of Scotland. What is more, blended whisky is formed as a mixture of different varieties of a single malt whisky during the production process, which may have an impact on the large variability of the data.

### 2.4. Semi-Quantitative Multivariate Data Analysis

Principal Component Analysis (PCA) was used as a primary method of data set evaluation in reference to the origin and type of the sample. PCA is variables reduction technique and sometimes is mistaken as the same statistical method. It is a method which can be applied for exploratory or confirmatory purposes. In our case, the correlation matrix (sums of squares and cross products from standardized data) was applied. It is normally used if the variances of individual variables differ much, or if the units of measurement of individual variables differ.

First stage of the interpretation of the results was the analysis of the main statistic parameters. The biggest mean values were recorded for three isotopes: ^48^Ti, ^54^Fe and^138^Ba. At the same time the highest variation of the results was observed for the following isotopes: ^45^Sc, ^47^Ti, ^48^Ti, ^52^Cr, ^63^Cu, ^66^Zn and ^138^Ba. Then the correlation among the studied variables was tested and highest relationship for some pairs of elements based on the PCA correlation matrix, was confirmed such as: ^47^Ti and ^48^Ti (0,99), ^47^Ti and ^138^Ba (0,92), ^209^Bi and ^208^Pb (0,91), ^48^Ti and ^138^Ba (0,90), ^47^Ti and ^90^Zr (0,89), ^138^Ba and ^90^Zr (0,89), ^48^Ti and ^90^Zr (0,87), ^44^Ca and ^45^Sc (0,87), ^60^Ni and ^138^Ba (0,84), ^111^Cd and ^90^Zr (0,80), ^118^Sn and ^66^Zn (0,80).

**Figure 4:**
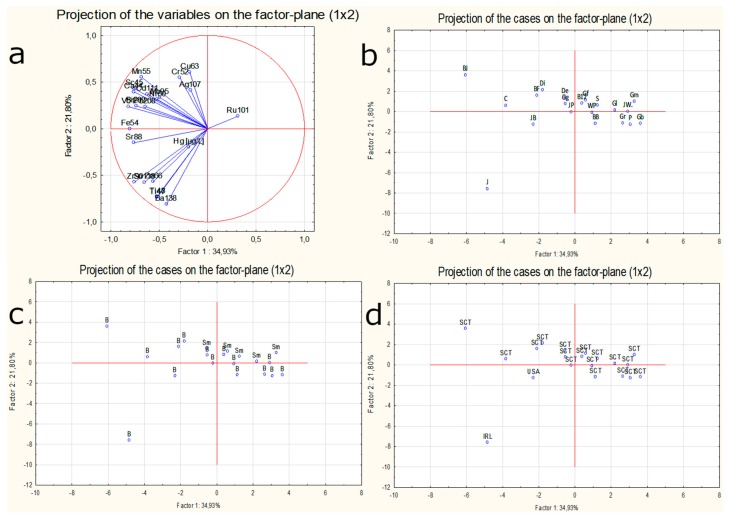
PCA analysis for variables (**a**) and objects (**b**,**c**,**d**) investigated in this study according to their type (**c**) and origin (**d**) for the whole data set including 22 variables: ^44^Ca, ^45^Sc, ^47^Ti, ^48^*Ti*, ^51^V, ^52^Cr, ^54^*Fe*, ^55^Mn, ^60^Ni, ^63^Cu, ^66^Zn, ^88^Sr, ^90^Zr, ^95^Mo, ^101^Ru, ^107^Ag, ^111^Cd, ^118^Sn, ^138^Ba, ^208^Pb, ^209^*Bi* (semi-quantitative data) and Hg [µg/L] (quantitative data) and 20 objects: whisky samples from the USA (Blended), IRL (Blended) and SCT (Blended and Single Malt) - all the samples details and abbreviations used in the graphs are explained in Table 1.

Performed PCA analysis revealed that first two components explained about 57% of the total variation (Figure 4). The principal components are correlated and explain less and less of the total variance of variables. Due the fact that after inclusion of the second main component other succeeding components did not clarify the whole variation of the data set only two first components were finally taken into account. Projection of the variables on the factor plane provided information about the correlation among the investigated isotopes in whisky samples. The strong correlation for e.g., ^47^Ti, ^48^Ti, ^138^Ba; ^45^Sc, ^44^Ca or ^118^Sn, ^90^Zr, ^66^Zn was verified. Almost lack of correlation was stated e.g., between ^55^Mn and ^138^Ba. The analysis of the input of each variable in the explanation of the variability of the whole data set proved that ^101^Ru, ^107^Au, and Hg have a insignificant influence on the first two components.

**Figure 5:**
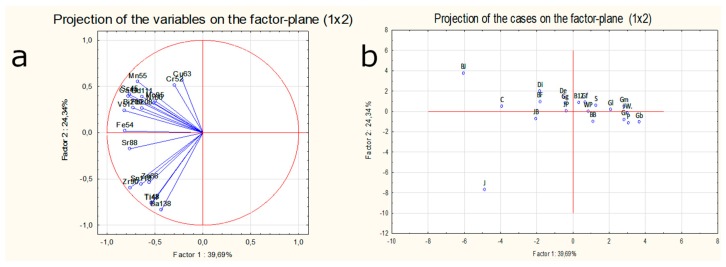

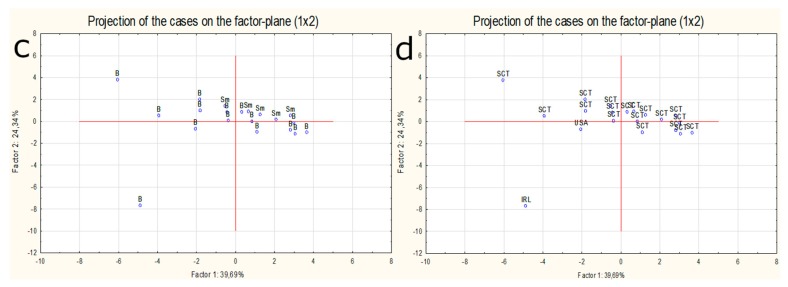
PCA analysis for variables (**a**) and objects (**b**,**c**,**d**) investigated in this study according to their type (**c**) and origin (**d**) after reduction of the number of a variables (after rejection of variables such as ^101^Ru, ^107^Ag and Hg), including 19 variables: ^44^Ca, ^45^Sc, ^47^Ti, ^48^Ti, ^51^V, ^52^Cr, ^54^Fe, ^55^Mn, ^60^Ni, ^63^Cu, ^66^Zn, ^88^Sr, ^90^Zr, ^95^Mo, ^111^Cd, ^118^Sn, ^138^Ba, ^208^Pb, ^209^Bi (semi-quantitative data) and 20 objects: whisky samples from the USA (Blended), IRL (Blended) and SCT (Blended and Single Malt)—all the samples details and abbreviations used in graphs are explained in Table 1.

Projection of cases on the factor plane for the first two components showed that all Single malt samples can be classified into one cluster. The blended type of whisky was much more dispersed on the factor plane. Samples analysed in reference to their origin demonstrated mostly four visible outliers on the factor plane: sample J (Irish whiskey), sample JB (USA burbon), sample C (Scottish whisky from the Speyside region) and sample BJ (Scottish whisky from Hinglands region). Sample BJ can be characterized by the highest amount ^45^Sc, ^51^V, ^52^Cr, ^54^Fe and quite high amount of ^44^Ca and ^88^Sr. It was not possible to discriminate Scottish whisky based on its origin, however, most of the scottish whisky can be grouped into one cluster with some exceptions (e.g., sample C, BJ). Definitely sample from USA (sample JB), and the sample from Ireland (sample J) can be clearly separated from the rest of the population. An attempt has been made to reduce the number of variables as well as the number of objects separately to verify the percentage of explained variation of the studied data set. For example, the rejection of variables such as ^101^Ru, ^107^Ag and Hg has not improved the overall percentage of solved variation (Figure 5). Even the reduction of the number of cases and rejection of the outliers like: C, J, JB, BJ has not resulted in clear classifications of a whisky sample in accordance with its region. Similar findings have been reported by other authors. Adam at al. concluded that the differences in Cu levels in whisky can be used to distinguish between Blended and Malt whisky but none of the concentrations of elements like Cu, Zn, Pb, Ni, Fe, Ca, Mg or Na can be successfully applied to identify various regions of whisky production in Scotland. Even with the use of cluster analysis no particular metal fingerprints were recognised for different geographical Scottish regions [18]. 

This thesis was supported by Shand et al., who based on the levels of 11 elements (P, S, Cl, K, Ca, Mn, Fe, Cu, Zn, Br, Rb) and PCA analysis unsuccessfully differentiated whisky in relation to their type or region of Scotland. In this paper however, the possibility of full separation of counterfeit products from original ones was stated [19].

## 3. Materials and Methods

### 3.1. Samples

Twenty whisky samples coming from various production regions of Scotland, Ireland and the USA were chosen for the elemental analysis. The types of alcohol collected for research are popular brands, widely available in stores. 

Studied samples varied both in terms of age (from three up to 18 years old) and type (Blended/Single malt). All information about the origin, type as well as age of the sample is summarized in Table 1. The whisky brands are coded according to their type and origin and codes are included in Table 1. The names of the manufacturing companies of the whisky are not given in this paper.

### 3.2. Samples Preparation and Measurement

Before the actual measurement with the use of the ICP-MS technique, it was necessary to mineralize the tested material. For this purpose, 2 mL of each whisky sample was measured with an automatic pipette and placed into glass tubes. Each sample was mineralized three times. Then 65% of nitric acid (V) (BAKER ANALYZED, Avantor Performance Materials Poland S.A., Gliwice, Poland, ultra-pure, with a maximum concentration of mercury below 5 ppb) was added in portions of 0.5 mL each. A blank test which did not contain alcohol was also prepared in the same way as the studied samples. The tubes were placed in the mineralizing unit (Ultrawave system, Milestone, Via Fatebenefratelli, Italy). After the mineralization process, the contents of the tubes were quantitatively transferred to class “A” flasks and filled with distilled water up to a volume of 25 mL. The mineralization process consisted of two steps:

- stage I (20 min): max. pressure inside the reactor 130 bar, max. temperature inside the reactor 230 °C and outside temperature 60 °C with a max. microwave power of 1500 W;
- stage II (10 min): max. pressure inside the reactor 130 bar max. temperature inside the reactor 230 °C and outside 60 °C with a max. microwave power of 1500 W.

A thick acrylic shield surrounds the working area, and correct and safe closure of the reactor is ensured by sensors located at multiple positions in the system. Unlike conventional microwave digestion systems, each sample is under direct temperature and pressure control. Temperature and pressure are controlled and displayed on the monitor with a frequency of 20 times/sec and the microwave power is automatically adjusted to control even highly exothermic reactions to ensure full control of the sample preparation procedure. In our study, given parameters of both stages mean that in any case the applied maximum power did not exceed 1500 W, but was successfully changed in accordance with the values of the acceptable temperature and pressure increase inside the reactor for each step within the established time.

Wet chemical analysis was performed by the ICP-MS technique for samples after the mineralization process. Before the semi-quantitative analysis by the ICP-MS technique, it was necessary to optimize the parameters of the mass spectrometer. The optimization process was performed based on the analysis of a solution with a known composition such as “Tuning Solution” containing elements such as: Li, Be, Bi, Ce, Co, In, Ba, Pb, Tl, U at a concentration of 10 μg/kg and covering the whole mass range. During optimization, special attention is paid to the signal from both oxides and double charged ions so that the ratio of both signals to the signal for the studied isotope does not exceed about 3%. During the optimization stage, the basic parameters are set routinely: The burner position, gas flows (load, additional and plasma), plasma power, lens voltages and the detector. For this purpose, signals are monitored for selected *m/z* ratios and the signal changes are monitored due to changes in the spectrometer settings. In this case, for each of three replicates of the same sample three spectra were collected. The acquisition time was 3 s and the average number of counts for selected isotopes was taken for further statistical analysis by Statistica 10.0 software (New York, NY, USA). 

In this study, no matrix reference material was employed. Due to the lack of any certificate reference material or any laboratory-made matrix material just before and just after the semi-quantitative measurements, the ICP spectra for the multi-element standard solution of Merck IV (ICP class) and for the sample blank (nitric acid which underwent the same mineralisation pre-treatment as the whisky samples) were recorded during a one-day analytical cycle. The diluted standard contained 30 elements (Ag, Al, As, B, Ba, Be, Bi, Ca, Cd, Co, Cr, Cu, Fe, Ga, K, Li, Mg, Mn, Mo, Na, Ni, Pb, Rb, Se, Sr, Te, Tl, U, V, Zn) at the concentration of 50 ppb for most of the elements. No significant drift of signal was observed for studied masses. Also no significant background increase connected with the use of the microwave oven system or chemical reagents was noticed. According to the Polish law, besides mercury content also the levels of three other elements, such as lead (max. 0.4 mg/kg), cadmium (max. 0.05 mg/kg) and arsenic (max. 0.20 mg/kg), are regulated. Based on the rough comparison of the intensity of signal for As, Cd and Pb in the studied samples and in the Merck VI standard solution in no case the admissible levels for these three elements were exceeded. Nevertheless, since no relevant standard reference material was commercially available, this work only focused on the multivariate analysis rather than the monitoring of some toxic metals. For both proposed statistical and chemometric approaches, there was no need to introduce fully quantitative data. Moreover, in this study, information regarding the intensity of the signal for the whole mass spectra with some minor exceptions was collected. All spectra for each whisky sample were scanned and compared to identify elements which can potentially discriminate the studied whisky among each other. However, the main idea for PCA analysis is to reduce highly correlated data in order to explain as much variance as possible since the additional information which is delivered by them is almost null. The noise information influences strongly the degree of the explained variation and in general introducing too much data without any selection can create problems with further data reduction. It is a well-known fact that the larger the variance, the larger the amount of information the variable contains. Thus, the primary elimination of some *m/z* ratios was necessary. The intensity of the signal for chosen *m/z* (raw data set) put into the algorithm was proposed in this study as a quick, screening method for possible whisky discrimination based on its type and origin. As a consequence, for the purpose of this study there was no need to quantify the exact levels of all elements included in this study since time-of-flight mass spectrometry offers a quasi-simultaneous analysis. The time-of-flight analyzer allows the user to separate ions about 400 times faster than a quadrupole analyzer, resulting in a shorter analysis time and lower sample consumption.The collection of the complete mass spectrum is possible in 25-30 μs (amounts 33.000 spectra/s), while for QMS the time interval is 10 ms. Thus, for this study it was sufficient to determine whether a specific element (for particular *m/z*) was present within a certain range and if there are any differences in the amount of this element within various whisky samples.

The automatic mercury analyzer does not require any special sample preparation stage. The proper volume of sample was injected into a ceramic boat and covered with the so-called additive B. The additive (active aluminum oxide) is typically used for samples which can also quickly evaporate at an early stage of thermal decomposition. Additionally, the additive can help to minimize the influence of the matrix. The ceramic boats and the additive were heated in the furnace at 680 °C before the analysis in order to eliminate the possible memory effect of mercury. Boats and additive were kept until analysis in the exsiccator.

Prior to the quantitative measurements of mercury content in the tested samples, it was necessary to create a calibration curve based on a mercury standard of 1000 mg/kg (Wako Pure Chemicals Industries, Takeda, Osaka, Japan) in a solution of l-cysteine acting as a stabilizer (Nacalai Tesque, Kioto, Japan). Calibration of the apparatus was carried out after preparation with subsequent dilutions of mercury standards containing: 0 ng Hg; 2 ng Hg, 10 ng Hg and 20 ng Hg (low ranges Hg) and 0 ng Hg; 10 ng Hg; 20 ng Hg; 50 ng Hg (high ranges of mercury). For this purpose, a small amount of additive “B” was introduced into ceramic boats. Next, using the automatic pipette, the proper volume of α-cysteine solution and α-cysteine solution containing Hg standard was introduced into the boats and in the end the samples were covered with the same amount of additive “B” as before. Three samples were measured for each amount of mercury. Whisky analysis was made in a similar way 50 μL of alcohol was introduced into each boat. 3 repetitions were conducted and the results were averaged.

### 3.3. Equipment

In a microwave system, the reactor is made of a robust stainless steel jacket to ensure that extremely high pressure conditions (up to 200 bar) and temperature (up to 300 °C) can be maintained. The tube with samples was placed in the Teflon container filled with water in order to assure the same conditions for each of the mineralized samples. The tubes were not tightly closed, only the Teflon lid was applied and the cover with the nitrogen gas under pressure of 40 bar [21].

The Mercury MA-3000 (Nippon Instruments Corporation, Tokyo, Japan) is a compact analyzer that allows traces of mercury to be measured in liquid or gaseous solid samples. The principle is based primarily on the thermal decomposition of the sample. Next, the mercury compounds break down and the disruptors are removed. In collecting tubes, which are also supposed to purify and condense, mercury is collected on a golden amalgam. After heating the tubes, mercury in the form of gas is released. Finally, the measurement is performed using atomic absorption at a wavelength of 253.7 nm. The automatic mercury analyzer has two measuring cells:

- an ultra-sensitive cell for small ranges of mercury (from 0.002 ng to 10 ng Hg),
- a cell for larger ranges of mercury (from 10 ng to 25,000 ng). Depending on the amount of mercury in the studied samples, the calibration range can be chosen for the quantitative analysis [22].

In this study, Inductively Coupled Plasma Mass Spectrometry with a time of flight analyzer was applied for the semi-quantitative study of whisky samples. The general ICP-MS technique is based on the generation of positively charged ions, which are separated according to the value of their mass to the charge ratio (*m/z*). Inside the time of flight analyzer ions are directed towards the drift tube, where they can be discriminated based on the difference of their speed by travelling the same distance. Thus, the obtained signal is time dependent and the detector (electron multiplier) records both the intensity and the time of flight of ions. All measurement conditions and parameters are included in Table 2. In our case, almost all mass spectra were collected ranging from 5 to 260 *m/z*, with some exceptions. For the statistical analysis, 21 isotopes were selected and verified if they can be applied for sample discrimination.

### 3.4. Data Analysis

The STATISTICA 10 software was used for statistical and multivariate analysis. The following methods of data interpretation were used in this work: box&whisker plots for all semi-quantitative and quantitative data, normality testing tests used for the verification of variables distribution, non-parametric tests for the verification of the influence of selected parameters on the semi-quantitative and quantitative data obtained for chosen *m/z*, principal component analysis (including the projection of the variables on the factor plane and the projection of the cases on the factor plane for the first two components).

➢ *Box and Whisker Plots*

The median was chosen to represent the central trend of results distribution and was labelled inside the frame in the diagrams as a small square. The results were divided into groups in which between 2 and 3 quartiles, by definition, 50% of all observations are located and the position of the frame on the graphs determines the position of the lower (2nd) and upper (3rd) quartiles. The width of the frame informs us about the diversity of 50% of the most typical units. The outliers were plotted as fences (whiskers) and were extended down to the minimum value (the length describing the variability of 25% of the lowest observations) and up to the maximum value (where the length was limiting 25% of the highest observations). These plots are very helpful in the visual presentation of the influence of selected factors on the distribution of studied elements. In our work, two main parameters were examined, namely the origin of the whisky product and the type of whisky (blended/single malt). 

➢ *Normality Tests*

Before the data interpretation, the verification of the normality of data distribution was necessary due to the fact that many statistical tests require the normal distribution of observations. They support in many ways the graphical assessment of the normality. In our case, three most popular tests were employed such as Kolmogorov-Smirnov (K-S), Lilliefors corrected K-S test and Shapiro-Wilk test. They all compare the scores in the sample to a normally distributed set of scores with the same mean and standard deviation. According to those tests, the null hypothesis makes the assumption that the sample distribution is normal. The graph of the probability function of such a normal distribution forms the shape of a Gaussian curve, where 68% of results are in the distance not exceeding 1 standard deviation from the average value and 95% of results—from two deviations. Obtaining results greater than the value of 3 standard deviations from the average is negligible and it can be assumed that this applies to approx. 0.2% in relation to the whole set of observations.

➢ *Test Interpretation*

When verifying any statistical hypotheses, the level of significance α is compared with probability p. The level of significance is nothing else than the assumed probability of making a mistake (otherwise an acceptable risk of error). Often, probability p is considered to be an extent to which the result is true, how representative it is to the entire population studied. This value was in our case set as α = 0.05. In the verification of statistical hypotheses, α was compared with the probability value of the test p and if p > α, then there was no reason to reject the *so*-called null hypothesis (eg the distribution of a given variable is a normal distribution). 

For the testing of the normality of distribution, the following 22 variables were included: ^44^Ca, ^45^Sc, ^47^Ti, ^48^Ti (isobaric interference from ^48^Ca), ^51^V, ^52^Cr, ^54^Fe (isobaric interference from ^54^Cr), ^55^Mn, ^60^Ni, ^63^Cu, ^66^Zn, ^88^Sr, ^90^Zr, ^95^Mo, ^101^Ru, ^107^Ag, ^111^Cd, ^118^Sn, ^138^Ba, ^208^Pb, ^209^Bi (semi-quantitative data) and Hg [µg/L] (total mercury content). On the basis of the conducted normality tests, the hypothesis of the normal distribution for most of the studied variables (*m/z*) should have been rejected (for the assumed significance level α = 0.05 significance level p < α), except for the results obtained for the ^45^Sc, ^51^V, ^52^Cr, ^54^Fe (an isobaric interference from ^54^Cr, ^55^Mn, ^60^Ni and ^88^Sr. Due to the lack of fulfilment of the normal distribution assumption, the non-parametric test such as Kruskal-Wallis was used. This test does not require equality with respect to variance in studied groups.

## 4. Conclusions

(1) The highest average intensity of the signal among all analyzed isotopes for studied whisky samples was recorded for titanium (**^48^Ti**) and barium (**^138^Ba**). Therefore, these elements play a key role in distinguishing Irish whisky from the rest. Moreover, for this sample also the highest intensity of signal for isotopes like: **^66^Zn, ^88^Ti**, **^90^Zr, ^118^Sn** was observed. All these isotopes create a characteristic fingerprint of Irish—made whisky.
(2) The use of multivariate analysis was crucial for the discrimination of samples based on their origin and type. The projection of cases on the factor plane for the first two components revealed that the sample of Irish whisky was an evident outlier. On the other hand the projection of the cases of the factor plane for the first two components proved that the blended type of whisky was more dispersed in this diagram, while the single malt type of samples was grouped within the same cluster. Within the studied population the scatter of results for most of the (with in exception of: ^63^Cu, ^101^Ru) analyzed isotopes for blended types of whisky was much more visible than for single malts. The dispersion of the results for both types of whisky can also be a consequence of the unequal number of valid cases.
(3) In any case the mercury content in analyzed whisky samples does not exceed the allowed level of 20 μg/L (according to the Polish Regulation of the Minister of Health concerning of the maximum level of mercury in spirit products containing more than 20% of alcohol).
(4) Future studies are needed in which quantitative data will be presented for a bigger number of objects originating from different regions of Scotlad and from the same distillery in order to evaluate if the analyzed bottle is representative for a particular brand.

## Figures and Tables

**Table 1:** The characterization of the whisky samples.

Sample Code	Type	Age	Origin
C	Blended (B)	12	Scotland-Speyside (SCT S)
BJ	Blended (B)	3	Scotland-Highlands (SCT H)
WP	Blended (B)	3	Scotland-Speyside (SCT S)
J	Blended (B)	5	Ireland (IRL)
BF	Blended (B)	3	Scotland-Highlands (SCT H)
B12	Blended (B)	12	Scotland-Highlands (SCT H)
BB	Blended (B)	3	Scotland-Highlands (SCT H)
Gg	Blended (B)	3	Scotland-Speyside (SCT S)
Gb	Blended (B)	3	Scotland-Speyside (SCT S)
Gr	Blended (B)	3	Scotland-Speyside (SCT S)
JP	Blended (B)	3	Scotland-Highlands (SCT H)
JB	Blended (B)	6	United States of America (USA)
P	Blended (B)	3	Scotland-Speyside (SCT S)
JW.	Blended (B)	3	Scotland-Lowlands (SCT L)
S	Single malt (Sm)	15	Scotland-Speyside (SCT S)
Gf	Single malt (Sm)	18	Scotland-Speyside (SCT S)
Gl	Single malt (Sm)	12	Scotland-Speyside (SCT S)
Gm	Single malt (Sm)	10	Scotland-Speyside (SCT S)
Di	Blended (B)	15	Scotland-Lowlands (SCT L)
De	Single malt (Sm)	12	Scotland-Highlands (SCT H)

**Table 2:** Information on the inductively coupled plasma-mass spectrometry (ICP-ToF-MS) OptiMass 8000, GBC measurement conditions/parameters.

ICP–ToF–MS	
Radio frequency power generator [W]	1350
Torch	Quartz
Nebuliser	Concentric quartz
Carrier gas	Argon
Spray chamber	Cyclonic
Plasma gas flow rate [L·min^−1^]	10
Auxiliary gas flow rate [L·min^−1^]	0.9
Nebulization gas flow rate [L·min^−1^]	0.76
Number of replicates	3
Acquisition time [s] Sampler Cones Sampler and Skimmer Cones	3 Ni Ni/Cu
